# Dual mechanisms governing reward-driven perceptual learning

**DOI:** 10.12688/f1000research.6853.1

**Published:** 2015-09-10

**Authors:** Dongho Kim, Sam Ling, Takeo Watanabe

**Affiliations:** 1 Department of Psychological and Brain Sciences, Boston University, Boston, MA, USA; 2 Center for Computational Neuroscience and Neural Technology, Boston University, Boston, MA, USA; 3 Donders Institute for Brain, Cognition and Behavior, Radboud University, Nijmegen, Netherlands; 4 Department of Cognitive, Linguistic, and Psychological Sciences, Brown University, Providence, RI, USA

**Keywords:** Perceptual learning, Vision, Reward, goal-directed, contingency, temporal contiguity, automatic, task-irrelevant

## Abstract

In this review, we explore how reward signals shape perceptual learning in animals and humans. Perceptual learning is the well-established phenomenon by which extensive practice elicits selective improvement in one’s perceptual discrimination of basic visual features, such as oriented lines or moving stimuli. While perceptual learning has long been thought to rely on ‘top-down’ processes, such as attention and decision-making, a wave of recent findings suggests that these higher-level processes are, in fact, not necessary.  Rather, these recent findings indicate that reward signals alone, in the absence of the contribution of higher-level cognitive processes, are sufficient to drive the benefits of perceptual learning. Here, we will review the literature tying reward signals to perceptual learning. Based on these findings, we propose dual underlying mechanisms that give rise to perceptual learning: one mechanism that operates ‘automatically’ and is tied directly to reward signals, and another mechanism that involves more ‘top-down’, goal-directed computations.

## Introduction

Perceptual learning is the process by which sensory systems in humans or animals improve their ability to perform a perceptual task, often after extensive experience with a particular stimulus. It had long been believed that this type of learning was tied to one’s task performance on that stimulus. Support for this came from a number of studies that found benefits of perceptual learning for features that were relevant to a task, whereas features that were merely exposed showed little-to-no learning ^1– 3^. Taken together, these studies supported the hypothesis that conscious effort directed toward a sensory feature, by means of processes such as attention, is necessary for the feature to be learned ^2– 4^.

In recent years, however, evidence for a new type of perceptual learning has emerged – one that may not necessitate higher-level, goal-directed processes, such as attention ^5– 14^. In a study by Watanabe *et al.* (2001), evidence was found that perceptual learning could transpire outside of the window of attention. Specifically, observers were asked to perform a demanding task at the center of a display, while they were exposed to an array of moving dots presented in the periphery. Importantly, only 5% of the dots moved coherently in a fixed direction, while the remaining dots moved randomly. Because the motion signal was task-irrelevant, it was assumed that little-to-no attention was actively deployed to that stimulus. Moreover, sensitivities to the 5% and 10% coherent motion were measured before (pre-test) and after (post-test) a training period. The strength of the 5% coherent motion was so weak that subjects were not able to discriminate or detect the coherent motion direction above chance, either at the pre-test or the post-test. Nevertheless, the result of the post-test revealed that repeated exposure improved sensitivity for the 10% coherent motion in the exposed direction. The authors interpreted these results as evidence for a new type of perceptual learning, coined ‘ *task-irrelevant perceptual learning*’, which occurs without attention ^5– 7^.

Is mere task-irrelevant exposure to a stimulus truly sufficient for perceptual learning? A follow-up study demonstrated that mere exposure is, in fact, insufficient; performance benefits of exposure only occurred when there was a *temporal pairing* between a task-irrelevant motion signal and task-relevant targets ^9^. Most interestingly, task-irrelevant learning appeared to only occur in instances in which the target was *successfully recognized* ^15^. Why is that? One interpretation is that successful recognition of the target letter led to a sense of accomplishment for the participant, which elicited an internal reward signal. As a consequence, task-irrelevant perceptual learning may arise as a result of repeated pairing between a stimulus and internal reward signals, which are released diffusively throughout the brain, affecting both task-relevant and task irrelevant stimuli ^5^.

Although the aforementioned studies did not *explicitly* test this reward-based hypothesis, a number of studies have since emerged, derived from work in animal models and humans, supporting the hypothesis that reward signals are sufficient in order for perceptual learning to manifest. In this article, we will review and synthesize work that has examined how reward signals play a role in shaping perceptual learning. Many models in cognition assume that goal-directed behavior plays a dominant role in governing learning. Goal-directed behavior is a class of behavior aimed towards completion of a task – a subset of self-attributed motives commonly assumed to require high-level cognitive processes, such as attention and decision-making. For instance, a classic example of a goal-directed behavior is the online computation of the probabilistic contingency between the presence of a stimulus, and receiving a reward. In phenomena such as reward-driven perceptual learning, an individual’s estimation of the ‘contingency’ between rewards and visual stimuli has been shown to impact learning rates, clearly indicating that goal-directed processes are involved. However, not all behaviors necessarily tap into these high-level processes. For instance, reward-driven perceptual learning has also been shown to occur in the absence of any task, as well as outside of an individual’s awareness. Interestingly, this suggests that reward signals can gate the emergence of learning, untainted by other higher-level cognitive processes. To explain these results, we propose dual underlying mechanisms of reward-driven perceptual learning: one mechanism that operates ‘automatically’, free from goal-directed processes, and another mechanism that involves more ‘top-down’, goal-directed computations, and requires conscious estimation of learning contingencies. Moreover, we propose that perceptual learning, in combination with paradigms used to suppress images from visual awareness, can be leveraged as tools to probe this more ‘automatic’ component of learning.

## How do rewards shape perceptual learning, independent of goal-directed processes?

The reward-driven hypothesis for task-irrelevant perceptual learning is based on the assumption that internal reward signals are released when subjects successfully recognize a target item, with the temporal pairing between a task-irrelevant feature and the reward signals playing a crucial role in determining task-irrelevant perceptual learning. However, it is possible that the task-based component in those aforementioned studies is unnecessary, and that it truly is the reward signal itself that triggers task-irrelevant perceptual learning. How does one test this hypothesis? The lion’s share of perceptual learning studies employ a training procedure by which observers perform a task that is the same or similar to evaluating the amount of learning. However, this makes it difficult to truly understand the effects of reward on perceptual learning, because the role of rewards in such paradigms is necessarily entangled with higher-level cognitive processes, such as attention and goal-directed decision-making, when participants are consciously performing a task on a stimulus. In order to truly understand how rewards gate perceptual learning, one should empirically disentangle rewards process from other cognitive processes. Classical conditioning is a process by which learning is acquired through repeated pairings of a stimulus and a reinforcer ^16^. Interestingly, classical conditioning does not necessitate any task during conditioning. Therefore, by leveraging classical conditioning, one can gain a true understanding of how reward signals modulate perceptual learning, untainted by goal-directed decision processes.

In a definitive test of the reward-signal hypothesis for perceptual learning, Seitz, Kim & Watanabe (2009) discovered that perceptual learning could occur even without any task involvement whatsoever. To do so, this study used a classical conditioning procedure in which human subjects, who were deprived of food and water, passively viewed visual stimuli while receiving occasional drops of water as rewards ^17– 19^. To ensure that perceptual learning was driven purely by reward signals, this study used a technique known as continuous flash suppression ^20^ throughout the training regime, which is known to render visual stimuli imperceptible. Surprisingly, learning occurred through stimulus-reward pairing in the absence of a task and without awareness of the stimulus presentation. Since neither task nor attention was involved during the training procedure, these results study strongly implicate the continuous temporal pairing between stimulus feature and reward signals as being the necessary and sufficient elements needed for perceptual learning at least in some conditions to occur.

## How do rewards shape perceptual learning, in the presence of goal-directed processes?

While reward signals alone appear to be sufficient to trigger perceptual learning ^10^, this result does not preclude goal-directed, conscious behavior from also playing a modulatory role in perceptual learning. According to the theory of motivation, implicit motives represent a more primitive motivational system derived from affective experiences ^21^, and it is likely that the task-irrelevant perceptual learning that has been observed rides on this motivational system to yield its effects. However, behavior is also driven by self-attributed (or explicit) motives, which are based on more cognitively elaborated constructs. Such goal-directed behavior, which requires higher-level cognitive processes, likely also governs perceptual learning. Indeed, it has long been suggested that such higher-level cognitive processes, such as attention and/or decision making, also act as main factors in driving perceptual learning ^1, 22– 25^.

What roles, then, do goal-directed behaviors play in perceptual learning? Specifically, do reward signals and goal-directed decision processes elicit a similar pattern of effects on perceptual learning? To examine the similarity between these ‘automatic’ and ‘top-down’ processes in perceptual learning, a recent study developed a methodology that combines perceptual learning with a novel training procedure, which employs either *classical conditioning* or *operant conditioning*. In the classical conditioning variant of the study, human subjects, who were deprived of food and water, passively viewed visual stimuli while receiving liquid rewards during a ‘training regime’ ^10, 17– 19^. This experiment was similar to the aforementioned study ^10^, with the notable exception being that there were various reward-contingencies at play, with the orientation content of a visual stimulus paired with a certain probability of receiving a liquid reward ^26^. To vary the probability of reward-delivery, three different stimulus orientations were used for each subject: 1) the zero-contingency orientation had a reward-probability equal to the background reward-rate of 50%, 2) the positive-contingency orientation had an 80% probability of reward, and 3) the negative-contingency orientation had a 20% probability of reward. In the operant conditioning variant of the study, a goal-directed behavior component was added ^27^. In contrast to the classical conditioning variant of the experiment, here subjects performed a ‘go/no-go task’ in response to the orientation stimuli during a training regime. Specifically, if subjects pressed a spacebar, a liquid reward was delivered at a probability contingent on the orientation of that presented stimulus (for example, 80% for a stimulus tilted 135°, 50% for a stimulus oriented 75°, and 20% for a stimulus oriented 15°).

Results from the classical conditioning variant of perceptual learning showed that learning occurred for both the positive-contingency orientation stimulus as well as the zero-contingency orientation stimulus, but no significant change was found for the negative-contingency orientation stimulus. In contrast, results of the operant conditioning variant of perceptual learning revealed that learning only occurred for the positive-contingency orientation, with no learning found for either the zero-contingency orientation or the negative-contingency orientation ^27^. These results suggest that reward-driven perceptual learning without goal-directed processing is distinct from reward-driven perceptual learning with goal-directed processes.

When there is no goal-directed behavior, a consistent pairing between a visual stimulus and reward seems to be the underlying mechanism for perceptual learning to occur ^5, 9, 10, 28^. In that case, “temporal contiguity” between rewards and visual stimuli play a crucial role for perceptual learning to occur ^5, 16^. However, if goal-directed processes are involved, contingency information between rewards and visual stimuli overrides pure temporal contiguity. In other words, the top-down component can override the automatic components of reward-driven perceptual learning. The operant conditioning variant of perceptual learning demonstrated that learning of a visual stimulus occurred only when that visual stimulus informatively predicted the upcoming rewards ^29– 32^.

These results square with a study by Law and Gold (2009), where monkeys carried out a goal-directed behavior, performing a visual task to receive rewards. In that study, connections between sensory neurons and the goal-directed decision process that interprets the sensory information were first modified by reward driven reinforcement signals. Subsequently, that same mechanism acted to further refine these connections to more strongly weight inputs from the most relevant sensory neurons, thereby improving perceptual sensitivity.

## Common mechanisms between perceptual learning and conditioning

Conditioning is the form of learning in which repeated pairings of arbitrary features with rewards or punishments leads to a representation of the rewards or punishment evoked by the paired features ^29, 33^. At face value, this resembles the task-irrelevant perceptual learning revealed in Seitz and Watanabe (2003), which occurred only when the visual feature was paired with the presentation of a rewarded target. A number of subsequent studies have demonstrated that task-irrelevant perceptual learning in humans can occur for visual stimuli that are consistently paired with internal or external rewards ^5, 9, 10, 28^, and this connection holds true for animal models as well ^34, 35^. Taken together, these studies suggest common mechanisms shared between conditioning and perceptual learning.

How generalizable are the rules governing conditioning to the domain of perceptual learning? One common theme to the perceptual learning and conditioning literatures is that of contingency ^29, 31, 36^. Excitatory conditioning occurs when the probability of a reward is higher for a conditioned stimulus than at other times, which is referred to as positive contingency. Likewise, when the probability is lower (negative contingency), negative conditioning occurs ^29, 31, 32, 37^. Since the contingency rule is a hallmark of conditioning, along with contiguity and prediction error ^29^, a question arises as to whether perceptual learning follows the same rules of contingency as found in conditioning. Were that the case, then one would expect to observe positive learning, negative learning, or no learning in accordance with the contingency between the predicted signal and the reward. Perceptual learning appears to be governed by both classical and operant conditions principles, depending on the situation. Under a task that promotes high-level processing of the stimulus-reward structure, perceptual learning mirrored the rules of operant conditioning, occurring only for the positive-contingency orientation, with no learning in either the zero-contingency orientation or the negative-contingency orientation ^27^. However, under a task that prevented high-level processing of the contingency structure, the effects of perceptual learning much more closely resembled learning of the ‘temporal contiguity’ between visual features and rewards in classical conditioning, with learning transfer occurring not only for the positive-contingency stimuli, but also for zero-contingency stimuli (of note, there were 50% stimuli-rewards pairings in zero-contingency stimuli). Although there has been considerable debate in regards to whether classical conditioning depends on a contingent relation between conditioned stimulus and unconditioned stimulus ^38^, perceptual learning under a task that prevented high-level processing of the contingency structure is more closely aligned with classical conditioning, in which learning is more influenced by contiguity than contingency ^39^.

## Conclusion

Perceptual learning can occur in the absence of a task and outside the window of awareness, suggesting that reward signals gate the occurrence of perceptual learning. This may emerge through mechanisms akin to classical conditioning, impinging on very early visual sensitivity. In that case, ‘temporal contiguity’ between rewards and visual stimuli plays a crucial role ^5, 16^. However, when goal-directed processes are introduced, the contingency between rewards and visual stimuli overrides classical condition-like operations, instead influencing perceptual learning based on the stimulus-reward contingencies. This suggests that there exists two underlying mechanisms that give rise to perceptual learning: one mechanism that operates ‘automatically’ and is tied directly to reward signals, and another overriding mechanism that involves ‘top-down’, goal-directed computations.
