# Supplementary material for: Dual mechanisms governing reward-driven perceptual learning
Source: F1000Res. 2015 Sep 10;4:764. [Version 1] doi: 10.12688/f1000research.6853.1 (PMC4629275; doi:10.12688/f1000research.6853.1)
Supplement: Peer review history

# Peer review: Referee response for version 1

**DOI:** 10.5256/f1000research.7372.r10916

**Published:** 2015-10-26

**Authors:** Leonardo Chelazzi

**Affiliations:** 1 Department of Neurological and Movement Sciences, University of Verona, Verona, Italy

I have greatly enjoyed reading this engaging and inspiring review article. The authors of the article provide a concise and highly focused review of the recent literature on visual perceptual learning, and notably of the so-called task-irrelevant perceptual learning (TIPL). The main thrust of the article is to draw a distinction between an automatic form of TIPL, whereby the repeated occurrence of reward in close temporal contiguity with a visual stimulus (feature) determines perceptual learning of that stimulus (feature), and a more complex and cognitively demanding form of perceptual learning, whereby perceptual learning occurs as a result of the task-dependent contingency between a given stimulus (feature) and reward.

I find this dichotomous view of perceptual learning, and of its modulation by reward, especially captivating and important, especially because we have recently elaborated a similar distinction between types of reward-dependent learning in the attentional domain that conform to classical conditioning and types that conform to operant conditioning ( Chelazzi *et al*., 2013). Similar to what elaborated here, in prior studies ( Della Libera *et al.*, 2011) we could detect an impact of reward on attentional learning that conformed to classical conditioning using task variants in which participants were led to believe that rewards were received independently of their performance, in a lottery-like fashion. Conversely, in other work ( Della Libera & Chelazzi, 2009) we could detect an impact of reward on attentional learning that conformed to operant conditioning using task variants in which participants were led to believe that rewards received in relation to specific stimuli depended on their attentional performance towards the same stimuli. I actually encourage the authors to note such remarkable consistency between notions that are being developed within the domain of perceptual learning and similar notions that are being developed within the domain of attentional learning. In this regard, it is important to eliminate any potential confusion. Perceptual learning refers to improvements in perceptual performance (increased sensitivity) in relation to specific stimuli (features) as a result of prolonged practice with/exposure to the stimuli (features). In contrast, attentional learning refers to an increase in the efficiency with which participants are able to select, or sometimes ignore, specific stimuli within multi-elements displays. It might well be the case that the two learning phenomena are intimately related, but such a possibility has never been formally tested, at least to the best of my knowledge.

One aspect that the authors of the present article might want to elaborate on further is the way in which they conceive the task goal-dependency of the perceptual learning effects necessitating top-down control. In operant conditioning, the key link between a given stimulus and reward is the behavioral response directed towards the stimulus. Reward can only be obtained to the extent that an instrumental response is produced towards the stimulus. Any contingency between the stimulus and reward is not sufficient to elicit operant conditioning. In keeping with this notion, in our own work (Chelazzi *et al*., 2013) we have proposed that in the operant conditioning-like context, the cognitive system is shaped by reward in such a way that particular attentional operations (either selection or suppression, which, in different terms, can be named prioritization or deprioritization) are reinforced in relation to specific stimuli. In this perspective, what is being learned is a specific cognitive act in relation to a given stimulus and the learning is guided by the reward contingency.

As a further development of the distinction put forward by the authors, I suggest that most likely the two forms of perceptual learning ought to differ in other important ways, including the resistance to extinction, the level of generalization across stimuli and tasks, and so forth.

One other aspect that the authors might want to consider is the extent to which the task-dependency of what they describe as the second and more complex form of perceptual learning actually requires that top-down control is exerted in relation to the stimulus for which learning is measured or whether for this type of learning to occur it is sufficient that top-down control is exerted in relation to any stimulus, not necessarily the one for which learning is measured. In the latter scenario, top-down control is engaged, though it is not directed towards the stimulus for which learning is measured, performance monitoring processes are at work, and any resulting reward is linked to ongoing performance. I suppose that this level of top-down control may be sufficient for the second type of perceptual learning to materialize.

Finally, Title and abstract are perfectly adequate.

I have read this submission. I believe that I have an appropriate level of expertise to confirm that it is of an acceptable scientific standard.

# Peer review: Referee response for version 1

**DOI:** 10.5256/f1000research.7372.r10747

**Published:** 2015-10-09

**Authors:** Wim Vanduffel

**Affiliations:** 1 Athinoula A. Martinos Center for Biomedical Imaging, Massachusetts General Hospital, Charlestown, MA, USA

This is a very well-written review aimed to provide mechanistic explanations for different variants of perceptual learning (PL). I enjoyed reading the manuscript and am inclined to follow the logic of the authors although a bit more data confirming the conjectures made by the authors would be desirable. The authors first review a number of papers from their laboratory that showed evidence for task-irrelevant perceptual learning. As opposed to more traditional forms of task-relevant PL, task-irrelevant PL occurs in the absence of attention and is supposed to be driven by external or internal rewards. Based on their data, the authors suggest the existence of two mechanisms giving rise to perceptual learning: an automatic reward-driven and a top-down goal-directed one. The studies listed in the review also suggest an interesting and common mechanism shared between conditioning and task-irrelevant perceptual learning.

I only have a number of suggestions that the authors may take into account to improve the manuscript.

1. The authors attempt to dichotomize mechanisms underlying task-relevant vs task irrelevant PL. Are these two parallel systems? Is it possible that only one mechanism exists which can be 'adjusted' based on task constraints? In other words, have they considered only one basic principle, whereby goal-directed PL is simply a variant of task-irrelevant PL? For example, one could argue that rewards are the driving force for perceptual learning dependent plasticity and that additional higher cognitive signals such as selective attention strengthen (or override) the reward-driven changes?
2. Given the fact that the authors promote two different mechanisms, it would be interesting if they can speculate about the neuronal pathways involved.
3. What should be done next? Are additional experimental demonstrations for task-irrelevant perceptual learning required? At this moment a rather limited set of stimulus parameters have been used in task-irrelevant PL paradigms. Does one need additional tests, for example by using other stimulus features, other sensory modalities? What about comparative experiments including other species? What about causal experiments trying to identify structures driving PL-dependent plasticity?

I have read this submission. I believe that I have an appropriate level of expertise to confirm that it is of an acceptable scientific standard.
